# Ultrapure laser-synthesized Si-based nanomaterials for biomedical applications: in vivo assessment of safety and biodistribution

**DOI:** 10.1038/srep25400

**Published:** 2016-05-06

**Authors:** Tarek Baati, Ahmed Al-Kattan, Marie-Anne Esteve, Leila Njim, Yury Ryabchikov, Florence Chaspoul, Mohamed Hammami, Marc Sentis, Andrei V. Kabashin, Diane Braguer

**Affiliations:** 1 Aix Marseille Université, INSERM, CRO2 UMR_S911, Faculté de Pharmacie, 27 boul. Jean Moulin, Marseille, France; 2 Aix Marseille Université, CNRS, LP3 UMR 7341, Campus de Luminy, 163 Avenue de Luminy, Case 917, 13288, Marseille Cedex 9, France; 3 Assistance Publique - Hôpitaux de Marseille, Hôpital Timone, 254 rue Saint Pierre, 13385 Marseille, France; 4 Service d’Anatomie et de Cytologie Pathologique, CHU Monastir 5000, Tunisie; 5 Aix-Marseille Université, CNRS, UMR 7263, Unité Chimie Physique, Prévention des Risques et Nuisances Technologiques, Faculté de Pharmacie, 13385 Marseille Cedex 5, France; 6 Laboratoire des substances naturelles, Institut National de Recherche et d’Analyse Physicochimique, Sidi Thabet, 2020 Tunisie; 7 National Research Nuclear University “MEPhI” (Moscow Engineering Physics Institute), International Laboratory “Bionanophotonics”,31 Kashirskoe sh., 115409 Moscow, Russia

## Abstract

Si/SiO_x_ nanoparticles (NPs) produced by laser ablation in deionized water or aqueous biocompatible solutions present a novel extremely promising object for biomedical applications, but the interaction of these NPs with biological systems has not yet been systematically examined. Here, we present the first comprehensive study of biodistribution, biodegradability and toxicity of laser-synthesized Si-SiO_x_ nanoparticles using a small animal model. Despite a relatively high dose of Si-NPs (20 mg/kg) administered intravenously in mice, all controlled parameters (serum, enzymatic, histological etc.) were found to be within safe limits 3 h, 24 h, 48 h and 7 days after the administration. We also determined that the nanoparticles are rapidly sequestered by the liver and spleen, then further biodegraded and directly eliminated in urine without any toxicity effects. Finally, we found that intracellular accumulation of Si-NPs does not induce any oxidative stress damage. Our results evidence a huge potential in using these safe and biodegradable NPs in biomedical applications, in particular as vectors, contrast agents and sensitizers in cancer therapy and diagnostics (theranostics).

Silicon is one of the most abundant elements on the earth, which is widely distributed in mammalian tissues in the form of orthosilicate (SiO_4_^4−^) and participates in numerous biological processes such as bone mineralization. Such exceptional biocompatibility of silicon gives a promise for its successful applications in various biomedical tasks^1^,^2^,^3^. However, the interaction of silicon-based nanomaterials with biological systems is quite different for pure Si nanoparticles and its most abundant compounds such as silicon oxide SiO_2_ nanoparticles (silica or glass nanoparticles). Despite a huge effort for last years on the employment of SiO_2_ nanoparticles as drug nanocarriers and other therapeutic agents, the existing data on their toxicity remain controversial^4^. Several *in vitro* studies suggest that mesoporous and colloidal SiO_2_-NPs do not affect cell viability at concentrations adequate for potential pharmacological applications^5^,^6^. Other studies evidence SiO_2_-NPs-mediated cytotoxicity which appears to be dose, time and size-dependent^7^,^8^. The data on *in vivo* toxicity of SiO_2_-NPs are even more contradictory^9^,^10^,^11^. One of problems is related to the fact that SiO_2_ nanoparticles are not biodegradable and they accumulate in various organs and particularly in liver, which causes a variety of detrimental effects, including Kupffer cells hyperplasia, hepatic inflammation, and oxidative stress, leading to changes in the biochemical composition of the liver^10^,^11^. Composed of Si nanocrystals covered by a thin silicon oxide SiO_x_ shell (1 <  x <  2) due to their interaction with the environment, Si nanoparticles or Si-SiO_x_ nanoparticles (Si-NPs) demonstrate much improved characteristics for biomedical applications^1^,^2^,^3^. Moreover, Si-NPs are not only biocompatible, but also biodegradable as in biological tissue they normally decay into orthosilicic acid Si(OH)_4_ that is naturally excreted from the body with the urine^12^. Park *et al.* observed a complete clearance of porous 126 nm Si-NPs four weeks after the intravenous administration of 20 mg/kg of Si-NPs in mice^12^. On the other hand, particular physico-chemical properties Si-NPs make possible a variety of imaging and therapeutic functionalities^1^,^2^,^3^. Si-NPs can be used as efficient drug delivery vectors^13^,^14^,^15^ and as contrast agents in imaging applications due to strong room temperature photoluminescence^16^,^17^. Si-NPs can also generate singlet oxygen and thus enable photodynamic therapy applications^18^,^19^. Finally, Si-NPs can be employed as efficient sensitizers of cancer hyperthermia under their illumination by infrared radiation^20^, radio frequency radiation^21^ and ultrasound^22^. However, despite prominent properties of chemically pure Si, Si-NPs are not always water-dispersible and typically contaminated by toxic products during conventional fabrication pathways, including solution-phase reduction^23^,^24^, microemulsion technique^25^,^26^, sonochemical synthesis^27^, mechano-chemical synthesis^28^, or post-fabrication treatments in solutions of acids^29^,^30^,^31^. Ultrasonic dispersion of electrochemically etched silicon^2^,^3^,^12^ is considered as a relatively clean method compared to others, but so prepared Si nanocrystals are still contaminated by hydro fluoric acid derivatives. In addition, strategies based on mechanical milling of porous Si matrix typically lead to a wide dispersion of both size and shape of Si NPs, which complicates their transport and stability *in vivo*.

Laser ablation in biocompatible solutions has recently emerged as a novel “physical” fabrication method, which promises a solution of the secondary toxicity problem of Si-based nanomaterials. In a common experimental setup, a Si target immersed in ultrapure deionized water is ablated by intense laser radiation, leading to a natural formation of Si nanoclusters^32^. When released to the aqueous environment, those nanoclusters coalesce and form a colloidal solution of spherical NPs, whose surface is free of any residual contamination. Although the ablation by conventional nanosecond lasers typically provides a broad distribution of interconnected Si nanostructures^33^,^34^,^35^,^36^, properties of laser-produced Si-based nanomaterials can be much improved by the employment of ultra-short (femtosecond) laser radiation, leading to much more uniform size characteristics, a large domination of crystalline phase and a drastically reduced tendency to agglomeration^37^,^38^. Furthermore, femtosecond laser fragmentation from microcolloids, preliminarily prepared by mechanical milling of a Si wafer, makes possible a fast production of concentrated solutions of low size-dispersed Si-based nanoparticles, while the mean size of nanoparticles can be controlled by varying initial concentration of Si microcolloids^21^,^39^. *Tamarov et al.* carried out first *in vivo* tests using laser-synthesized Si NPs and results of these tests were very encouraging^21^. First, low doses of Si-NPs produced by laser ablation (4.9 mg/kg) were not toxic to rat after oral administration, as no statistically significant changes in blood levels of aminotransferases, alkaline phosphatase, bilirubin and cholesterol were detected. The intravenous injection of dextran-covered Si-NPs (10, 20 and 30 mg/kg) also did not cause any side effects, as shown by viability tests in mice. It was also shown that laser-synthesized Si-NPs can serve as efficient sensitizers for radio frequency induced cancer therapy^21^ and photodynamic therapy^38^. Despite these first encouraging results, up to now there is no systematic toxicity data available for ultrapure Si-SiO_x_ NPs prepared by laser ablation in aqueous biocompatible solutions.

This paper is conceived as an attempt to clarify the interaction of ultrapure laser-synthesized Si-based nanoparticles with biological systems *in vivo*. We systematically studied the NPs cytotoxicity *in vitro*, as well as their safety, biodistribution and excretion *in vivo*. It is implied that this study will play a key role in the assessment of behavior of Si-NPs in biological systems and consequently will validate their use as a theranostic tool in cancer therapy tasks.

## Results

### Characterization

**Figure 1:**
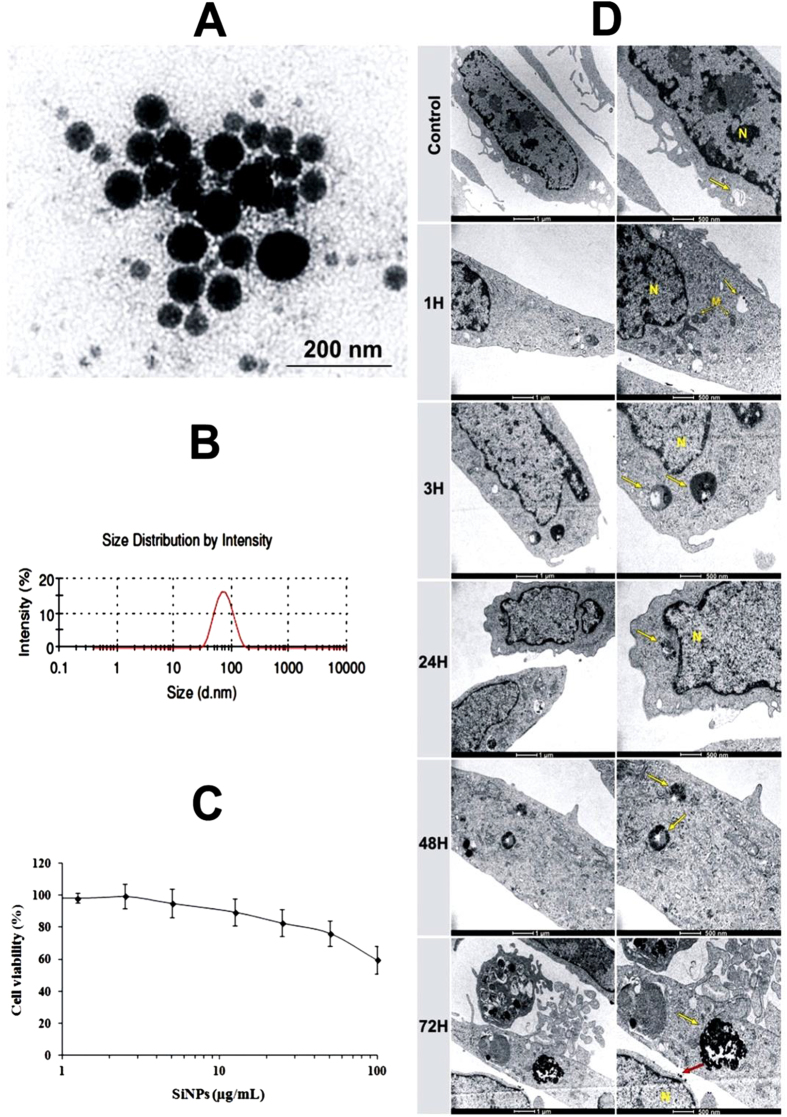
(**A**) TEM images of silicon nanoparticles (Si-NPs) fabricated by laser ablation in water and then transferred into NaCl 0.9% buffer. (**B**) Size distribution of Si-NPs in NaCl 0.9% determined by Zetasizer Nano ZS instrument (DLS). (**C**) MTT assays of HMEC cells viability following their exposure to different concentrations of Si-NPs (1.25–100 μ g/mL) for 72 h. (**D**) TEM images of HMEC cells showing kinetics of Si-NPs cell internalization studied 1, 3, 24, 48 and 72 h after incubation with 50 μ g/mL of Si-NPs. Si-NPs are inside lysosomes (yellow arrows). The right side is a magnification of the left side. (M =  mitochondria, N =  nucleus). Si-NPs remained attached to the cell membrane after 72 h of incubation (red arrows).

Si-NPs were fabricated by methods of femtosecond laser ablation in deionized water^38^,^39^ (see details in Methods section). The nanoparticles were dispersed in physiologic sodium chloride solution (NaCl 0.9%) for morphology and size distribution analysis. Transmission electron microscopy (TEM) and dynamic light scattering (DLS) measurements showed that the formed nanoparticles were spherical with the mean size around 50 nm and the size dispersion lower than 40 nm full-width-at-half maximum (Fig. 1A,B). Si-NPs were negatively charged, according to the zeta potential measurement (−35 mV).

### *In vitro* safety assays and the kinetic of Si-NPs cell internalization

Cell survival was quantified by using the MTT colorimetric assay, which reports on the metabolic activity of the cells mitochondria. Si-NPs having concentrations ranging from 1.25 to 100 μ g/mL were tested on both HMEC and RAW264.7 cell lines after 72 h of incubation. The cell viability profile of HMEC did not show any obvious cytotoxicity up to concentrations of 20 μ g/mL (Fig. 1C). The inhibition of cell survival below 25% was observed at high concentration of Si-NPs (50 μ g/mL), indicating a satisfactory low toxic effect. Similarly, the inhibition of cell survival was lower than 20% while Si-NPs having concentration of up to 100 μ g/mL were incubated in mouse monocyte-macrophage RAW264.7 cells (Fig. S1), confirming the absence of Si-NPs-induced redhibitory toxicity effects. Kinetics of Si-NPs cell internalization was studied by TEM 1, 3, 24, 48 and 72 h after HMEC cell incubation with 50 μ g/mL of Si-NPs (Fig. 1D). After 1 h of incubation, spherical Si-NPs were observed inside endosome vesicles (arrow). The amount of Si-NPs increased slowly inside endosomes until 48 h of nanoparticles incubation, suggesting time dependent cellular uptake of Si-NPs. The size of intracellular Si-NPs was ranged from 30 to 70 nm. The morphology of cell organelles including nucleus and mitochondria was normal compared to the control cells, indicating the absence of Si-NPs toxic effect until 48 h of incubation. After 72 h of incubation, a few cells subjected to a slight swelling were observed. Similar phenomena were observed using U87-MG cancer cells (Fig. S2).

### Mice behavior and growth

Four groups of 6 athymic nude female mice were intravenously injected with a single dose of 20 mg/kg of Si-NPs and then examined for survival 3, 24, 48 h and 7 days after the injection. For comparison, four control groups of athymic nude mice were intravenously injected with NaCl 0.9%. Mice body weight and behavior were monitored after Si-NPs administration to explore the presence of toxic effects due to the injection of Si-NPs. All animals showed healthy aspect and normal activity in the absence of lethargy or apathy after Si-NPs administration. In addition, normal fully open eyes with no secretion and totally normal breathing were monitored. No significant difference in body weights was noted for Si-NPs-treated animals, as compared to the control group (Fig. S3A). Here, during first 7 days the body weight of the mice injected with Si-NPs slightly increased in a pattern similar to the control mice, suggesting a normal mice growth in the absence of any significant toxic effect. Some animals (groups of 24 h, 48 h and 7 days) were placed in metabolic cages 24 h before the sacrifice in order to follow their food and water consumption and to recover urines and feces for further determination of silicon content. No significant difference was observed in water intake or food consumption (Fig. S3B,C), nor in urine volume or feces weight between treated and control groups (Fig. S4A,B).

### Biodistribution of Si-NPs

**Figure 2:**
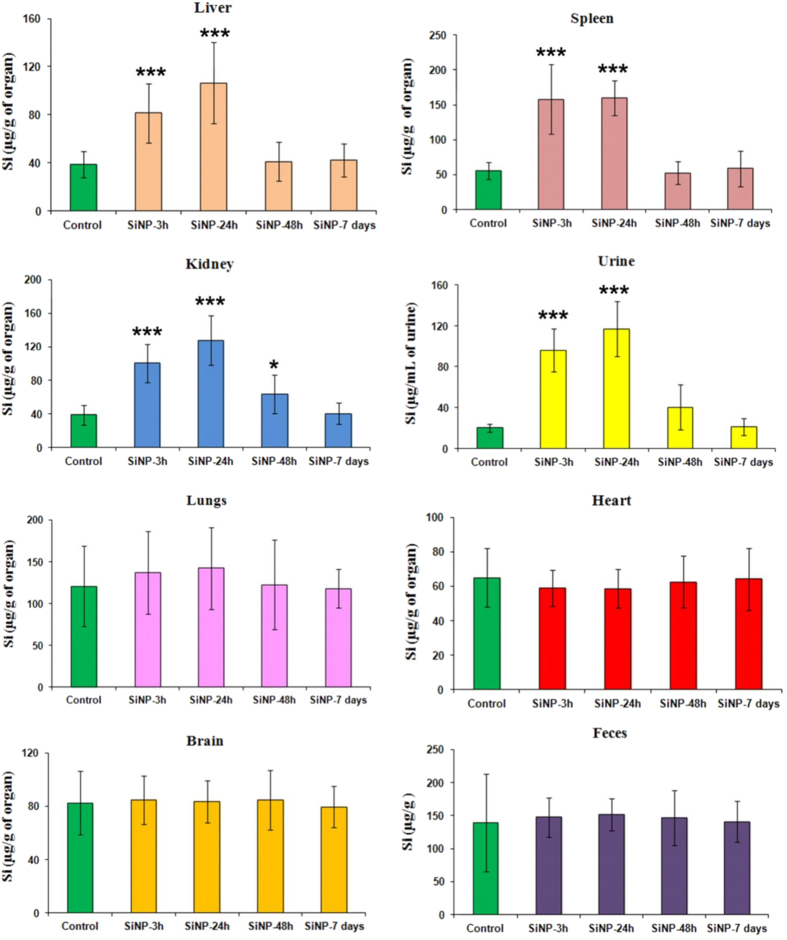
Silicon content in different organs, urine and feces of mice intravenously administered with a solution of Si-NPs (20 mg/kg) 3 h, 24 h, 48 h and 7 days after the injection related to control group of mice (n = 6, data are the mean ± SD). Statistical significance was determined by Student’s t-test. *p <  0.05, **p <  0.01, ***p <  0.001 compared to the control.

Biodistribution of Si-NPs was studied by quantifying silicon concentration in several complex biological matrices, including liver, spleen, lungs, heart, brain, kidneys, feces and urine. As shown in Fig. 2, silicon content differed from one organ to another, indicating a heterogeneous character of Si-NPs biodistribution. Here, no significant difference in silicon content compared to the control groups was observed for lungs, heart, brain and feces of all mice injected with Si-NPs. However, three-fold increase of Si content was recorded 3 h after Si-NPs injection in spleen (155,5 μ g/g) and after 24 h in liver (106.4 μ g/g), evidencing a rapid sequestration of Si-NPs by the reticuloendothelial system, notably by liver and spleen. At the same time, Si concentration increased significantly in kidneys and urine 3 h and 24 h after Si-NP injection reaching 127 μ g/g and 117 μ g/mL, respectively, and then decreased progressively after 48 h. 7 days after Si-NPs administration, silicon content came back to normal values in all matrices, as compared with the control groups. Finally, we determined the ratio of remaining Si-NPs to initially intravenously administered dose (20 mg/kg) for different organs, urine and feces at different moments after the NPs injection (Table 1). One can see that about 31 and 48% of the administered NPs were eliminated with the urine 3 h and 24 h after the intravenous administration of Si-NPs, respectively. It should be noted that Si content in feces, heart and brain were too low to be monitored. Combined together, these results demonstrated that Si-NPs were rapidly eliminated from the bloodstream, accumulated in liver and spleen, and then degraded and completely cleared from the body *via* urine within a period shorter than 7 days.

### Examination of organs for Si-NPs accumulation and degradation

**Figure 3:**
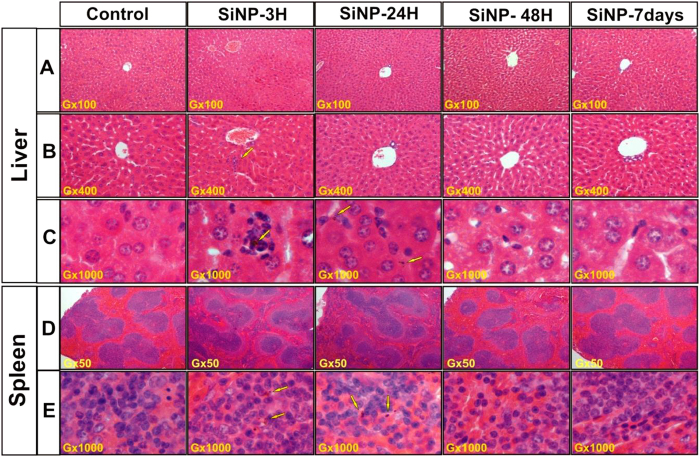
Histology of mice livers (**A–C**) and spleen (**D,E**) 3 h, 24 h, 48 h and 7 days after the intravenous administration of Si-NPs (20 mg/kg) or NaCl 0.9% (for the control group). Sections were stained with haematoxylin and eosin. The arrows indicate Si-NPs taken up by Kupffer cells in the liver and macrophages in the spleen. (**B,C**) are magnification of A. (**E**) is magnification of (D).

Macroscopic examination of all organs revealed a normal aspect and color, without hyperplasia or necrosis. Moreover, all mice livers showed a normal morphology without hypertrophy or adherent lobes (data not shown). A comparison of organ weights between treated and control groups did not show any statistically significant difference, as confirmed by student test (Fig. S5). Histopathological examination of liver tissues did not reveal any major negative effects, as it was observed under intravenous administration of SiO_2_ nanoparticles^10^,^11^. Here, we observed only very minor inflammation effects on hepatocytes, as revealed by normal parenchyma architecture without apparent changes in the hepatocytes structure. We also did not reveal any sign of hypertrophy or hyperplasia of perisinusoidal cells and observed only slight transient hepatic neutrophils infiltrations in livers 3 h after Si-NPs administration (Fig. 3A,B), suggesting a minor acute inflammation due to the rapid elimination of Si-NPs from the blood stream and their sequestration inside macrophages (Fig. 3). However, no sign of inflammation was detectable 48 h after the intravenous administration, evidencing a rapid degradation and removal of the nanoparticles. Si-NPs were clearly visible as brown clusters inside Kupffer cells, as evidenced by hematoxylin-eosin staining microscopic observations (Fig. 3C, arrows) taken 3 h and 24 h after the NPs administration. 48 hours after the injection these nanoparticles were not resolvable (Fig. 3C), confirming a rapid clearance of Si-NPs from the liver.

**Figure 4:**
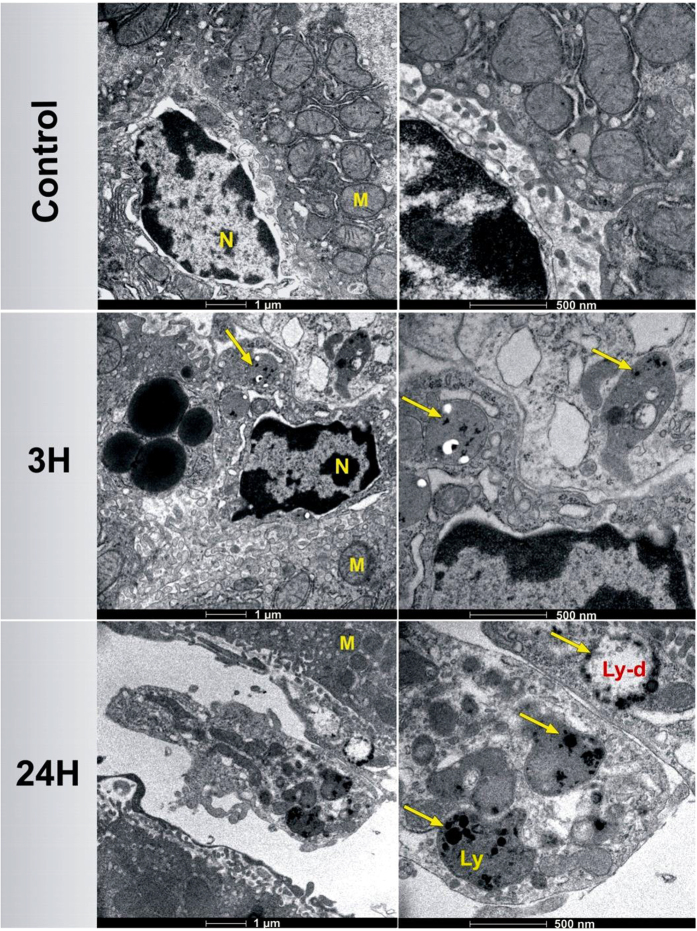
TEM images of mice livers 3 h and 24 h after the intravenous administration of Si-NPs (20 mg/kg) compared to the control group administered with NaCl 0.9%. Arrows show the presence of Si-NPs within the lysosomes (Ly) in Kupffer cells. Lysosomes-containing Si-NPs show a brighter area (Ly-d) around nanoparticles in liver 24 h after Si-NPs administration, suggesting an efficient degradation of Si-NPs under lysosomal conditions. (M =  mitochondria, N =  nucleus).

TEM images of liver slices were taken 3 h and 24 h after the NPs injection in order to precisely locate the position of Si-NPs within cell organelles. While an important number of accumulated Si-NPs were detected inside Kupffer cells (Fig. 4), the other types of cells (e.g. hepatocytes and endothelial cells) were totally free of Si-NPs. No sign of toxicity such as cytoplasm or nucleus degeneration was observed in Kupffer cells due to the accumulation of Si-NPs. A well-distributed nuclear chromatin in the condensed nucleus and a spherical or oval morphology of mitochondria indicated the absence of cellular toxicity. Moreover, Si-NPs with different sizes were observed inside lysosomes (Ly) as an electron-dense material with a brighter area (Ly-d) around nanoparticles subjected to the degradation process.

Histopathological examination of spleen tissues revealed an important macrophagic activity 3 h and 24 h after Si-NPs administration (Fig. 3D,E), which was characterized by the infiltration of macrophages (containing Si-NPs) in the marginal zone and in the red pulp, indicating an instant inflammation due to the accumulation of a large amount of Si-NPs. However, 48 h and 7 days after Si-NPs administration, the surface of both marginal zone and red pulp came back to the normal, as compared to those of the control spleen (Fig. 3D,E), indicating the disappearance of NPs-induced inflammation. Si-NPs were exclusively located inside the splenic macrophages similarly to the hepatic tissue, as was confirmed by microscopic examinations (Fig. 3E) and TEM observations (Fig. S6) of mice tissues 3 h and 24 h after the Si-NPs injection. Note that during first 24 h after NPs administration, macrophage lysosomes were filled with electron-dense accumulated Si-NPs (arrows).

**Figure 5:**
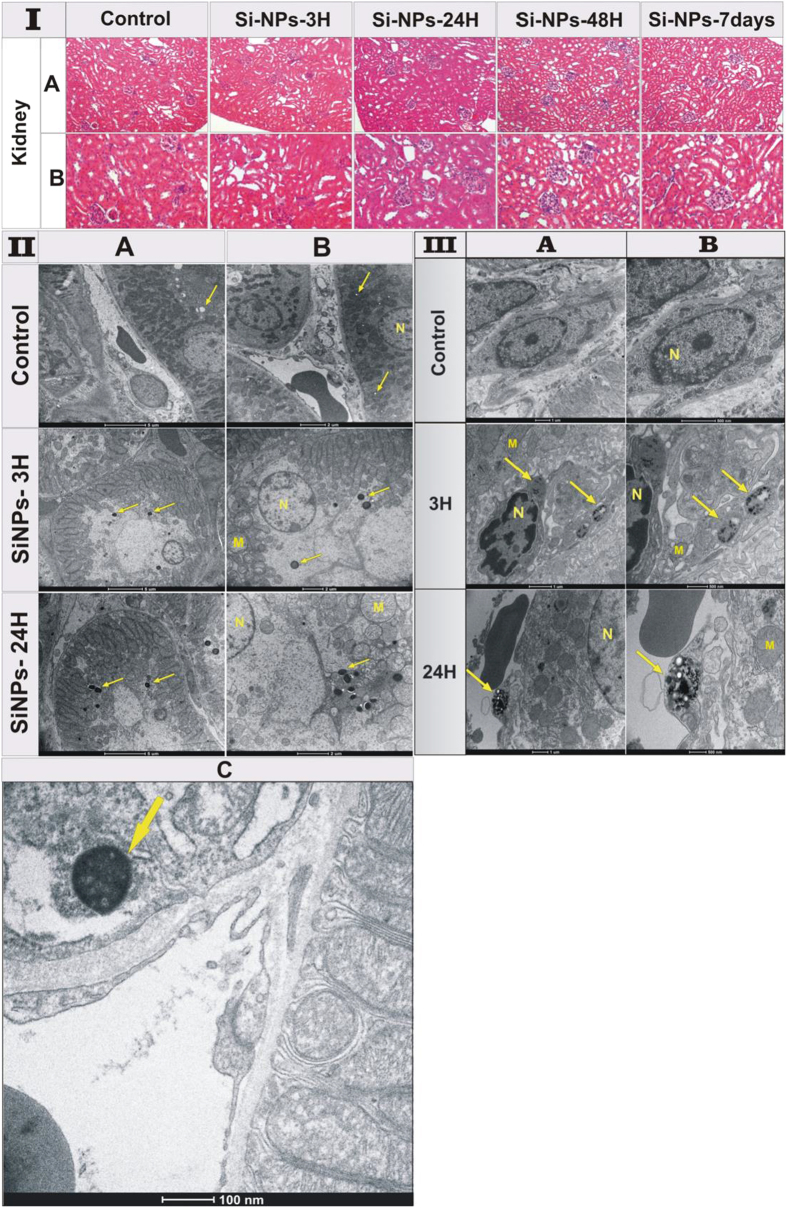
(**I**) Histological sections of mice kidneys 3 h, 24 h, 48 h and 7 days after the intravenous administration of Si-NPs (20 mg/kg) compared to the control group administered with NaCl 0.9%. All sections were stained with hematoxylin–eosin. **(II)** TEM images of kidneys of the control mice and treated mice 3 h and 24 h after Si-NPs administration. Si-NPs with size less than 6 nm were filtrated by glomerular capillary and then accumulated inside proximal tubular lysosomes indicated by arrows, (**A**) the scale bar is 5 μ m, (**B**) the scale bar is 2 μ m. (**C**) magnification of kidney of mice sacrified 24 h after Si-NPs administration, showing the accumulation of Si-NPs inside lysosomes (arrow) of the proximal renal tubules prior to urine excretion. **(III)** TEM images of mice kidneys 3 h and 24 h after the intravenous administration of Si-NPs (20 mg/kg) compared to the control group administered with NaCl 0.9%. Si-NPs with sizes larger than 6 nm are captured and accumulated inside lysosomes indicated by arrows. (M =  mitochondria, N =  nucleus). (**A**) is the magnification of (**B**).

It is important that the amount of nanoparticles in liver and spleen progressively decreased 24 h after the NPs injection and completely vanished 48 h after the injection. Such data were consistent with the increase of silicon content in kidney and urine, as confirmed by biodistribution results evidencing a complete degradation of Si-NPs and their elimination via urine. The histological examination of kidneys of Si-NPs-treated mice 3 h, 24 h, 48 h and 7 days after the NPs injection revealed normal tissue architecture related to the control groups (Fig. 5I). Indeed, kidney tubule tissue of all animals showed normal appearance of the tubular epithelium. In addition, the renal cortex appeared without histological alteration such as cytoplasmic vacuolization, proliferative glomerulonephritis or membranous glomerulopathy.

Finally, TEM images of renal epithelium of Si-NPs-treated mice for 3 and 24 h showed a normal brush border, basement membrane, and intact cell organelles, including mitochondria, nuclei, and a few membranous vacuoles (Fig. 5II). Here, we could observe numerous normal long-shaped mitochondria between extensive infoldings of basolateral plasma membrane creating a lateral cell processes, and a prominent lysosomal compartment containing Si-NPs as electron condensed nanomaterial (Fig. 5II,A,B,C, arrows). This classical ultrastructure of kidney supported the lack of toxicity due to the accumulation of Si-NPs inside lysosomes of the proximal renal tubules prior to urine excretion. Note that for kidney of the control groups, lysosomes were empty of electron condensed nanomaterial (arrows). It is important that no renal damage including acute tubule necrosis, interstitial nephritis or renal failure was observed. Interestingly, Si-NPs could be observed as accumulated condensed material inside kidney macrophages (Fig. 5III), suggesting the capturing of Si-NPs with sizes larger than 6 nm due to the impossibility of crossing the glomerular filtration. Finally, histopathological examination of the rest of organs including the lungs, heart and brain did not reveal any histological alteration or accumulation of Si-NPs (Fig. S7), as was confirmed by monitoring of silicon content in these organs.

### Biochemical parameters

**Figure 6:**
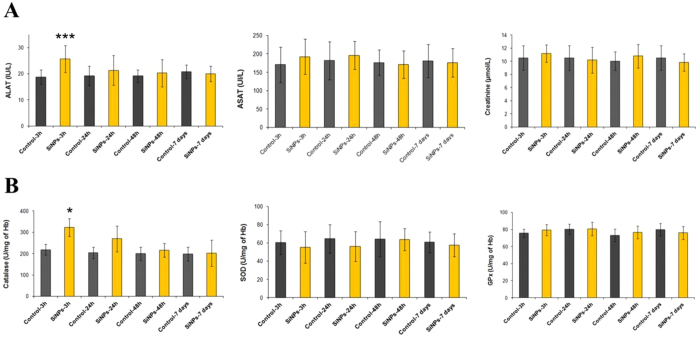
(**A**) Serum alanine aminotransferase (ALAT), aspartate aminotransferase (ASAT), and creatinine levels (**B**) blood catalase, superoxide dismutase and glutathion peroxidase activities determined in control and treated mice with 20 mg/kg of Si-NPs 3 h, 24 h, 48 h, 7days after the intravenous administration of Si-NPs. Data are the mean ±  SD, (n =  6) and statistical significance was determined by Student’s t-test (*p <  0.05, ***p <  0.001).

The accumulation and clearance of NPs in organs can potentially cause their damage. To control these processes, we monitored activities of typical biochemical markers of hepatic cytolysis (serum alanine aminotransferase (ALAT), aspartate aminotransferase (ASAT), and kidney function (serum creatinine level). In addition, we controlled oxidative stress markers, including blood catalase, superoxide dismutase, glutathione peroxidase, and serum vitamins A and E. As shown in Fig. 6, ALAT and catalase activities increased significantly 3 h after Si-NPs administration and then came back to normal 24 h after the injection (as compared to the control groups). In contrast, activities of ASAT, superoxide dismutase, glutathione peroxidase and creatinine level remained totally normal after the Si-NPs administration. Moreover, the accumulation of Si-NPs and their biodegradation did not cause any significant variation on the levels of both vitamin E and A (Fig. S8). Here, no oxidized derivatives of vitamins E and A were detected and this was confirmed by HPLC analysis of serum extract (Fig. S8). Finally, we determined the serum level of Interleukin-6 (IL-6), which is a well-known mediator of inflammation. We found that IL-6 increased 3 h after intravenous administration of Si-NPs and then progressively decreased after 24 h to come back to its normal level 48 h after the NPs injection (Fig. S9). Such process was not accompanied by any granuloma formation despite the presence of aggregates of Si-NPs observed in Kupffer cells or in spleen macrophages (Figs 3, 4 and S5), suggesting the absence of chronic inflammation after Si-NP intravenous administration.

## Discussion

The assessment of nanoparticle toxicity in cells is a prerequisite of any *in vivo* studies. Size, surface charge or shape characteristics can affect the interaction of nanoparticles with biological compounds and thus condition their toxicity^40^. In our study, safety of Si-NPs was tested *in vitro* on healthy human microvascular endothelial cells (HMEC) and mouse monocyte-macrophage cells (RAW264.7). The inhibition of cell survival below 25% and below 20% was observed for HMEC and RAW264.7 cells, respectively, under relatively high concentrations of Si-NPs (50 μ g/mL and 100 μ g/mL, respectively), suggesting a satisfactorily low toxicity effect. Luminescent porous Si-NPs synthesized by electrochemical process (por-Si NPs) were previously tested at similar range of concentrations (1 to 100 μ g/mL) on cervix cancer HeLa cells and did not show any significant cytotoxicity after 48 h of incubation^12^. The reported difference in cytotoxicity for laser-ablated and electrochemically-synthesized Si-NPs is obviously due to different experimental protocols, as cell cultures were different and the incubation time of por-Si nanoparticles in ref. 12 was much shorter (48 h instead of 72 h). It should be noted that laser-synthesized Si-NPs with concentrations similar to this study (60 μ g/mL) were earlier tested with laryngeal cancer cells (Hep2) and did not show any cytotoxic effect after 24 h of incubation^21^.

As shown in TEM images (Fig. 1D, Fig. S2), Si-NPs remained attached to the cell surface 72h after their incubation, both for HMEC and U87-MG cell lines, indicating the saturation of nanoparticles uptake by cells or their exocytosis outside cell membrane similarly to what was observed in other studies^41^,^42^. We postulate that negative surface charge of Si-NPs (ξ potential ≈  − 35 mV) together with the hydrophobic nature of Si-NPs favored a strong nanoparticle-membrane interaction, but still prevented extensive internalization, as it was earlier reported for titanium and hydrocarbonized porous silicon nanoparticles^43^,^44^,^45^. In their study of interaction between magnetic inorganic nanoparticles and macrophages, Wilhelm *et al.*^46^ described the particle uptake kinetics as a two-step process: the first step is the binding of anionic magnetic nanoparticles onto the cell surface, which is described as a Langmuir adsorption, and the second one is cell internalization, which is described as a saturable mechanism. Saturation of cellular uptake of nanoparticles could be due either to the equilibrium between endocytosis and exocytosis^47^ or to the limited capacity of internalization/endocytosis of the cells^48^,^49^. Taking into account these data, we suggest that cellular uptake of Si-NPs occurs *via* endocytotic mechanism. Anyway, our results confirm complete safety of Si-NPs and evidence their high potential for biomedical applications.

*In vivo* studies also confirm very low toxicity of Si-NPs. Just after intravenous administration, the nanoparticles were rapidly sequestered by the reticuloendothelial system (RES), notably the liver and spleen. It is well known that liver is the major target of nanoparticles accumulation (mainly inside Kupffer cells) after the intravenous administration^50^,^51^. Si-NPs were rapidly coated by opsonins, which facilitated their recognition by macrophages and a rapid elimination from the bloodstream to accumulate in liver and spleen and then be eliminated^52^,^53^,^54^,^55^. We suppose that the accumulated Si-NPs were degraded under lysosomes conditions. This supposition is confirmed by TEM images (Fig. 4), which evidence the presence of bright area around nanoparticles in lysosomes of mice liver, similarly to what was observed with other nanoparticles types^56^. The biodegradation properties of Si-NPs provide a pathway for their safe clearance from the body. Our tests confirmed a rapid elimination of Si-NPs from the bloodstream, subsequent sequestration by the RES, degradation and elimination *via* urine 48 h after the NPs injection. A fast recovery of normal volumes of soft feces enabled us to confirm the absence of constipation phenomena, while the identical content of feces of Si-NPs-treated and control group mice suggested that Si-NPs were not eliminated through the bile ducts. In addition, TEM analysis of kidneys (Fig. 5) showed that 5–6 nm Si-NPs were rapidly cleared from the body via renal filtration and urinary excretion, as was confirmed by the presence of silicon content in urine matrix. Since the liver is the major route for nanoparticles degradation through metabolism function^57^,^58^, we suggest that under the harsh biological conditions (salinity, lysosomal pH and enzymatic activity) Si-NPs were biodegraded and then excreted with the urine via renal filtration. It is known that NPs with sizes smaller than 6 nm are able to cross glomerular filtration^59^. We believe that unfiltered Si-NPs (>6 nm) were captured by kidney macrophages to be either degraded into smaller nanoparticles or solubilized into orthosilicic acid by lysosome digestion. In both cases, Si-based residual products are excreted with the urine, as reported previously for luminescent porous Si-NPs^12^.

It is important that we observed a complete elimination of laser-synthesized Si NPs in a period shorter than 7 days after the injection, while most other nanomaterials demonstrate drastically slower clearance kinetics^10^,^11^,^26^,^60^,^61^. As an example, a significant portion of silica (SiO_2_) nanoparticles were detected in histological samples 30 days after their injection^10^,^11^. It is interesting that porous Si-based nanoparticles (por-Si) prepared by electrochemistry routes were also cleared much more slowly^12^ compared to laser-synthesized Si-NPs (4 weeks compared to 7 days). We believe that such a retarded elimination of por-Si NPs could be due to their larger size and irregular shape, as well as contamination of their surface by HF derivatives during the electrochemical synthesis. A rapid biodegradation of laser-synthesized Si/SiO_x_ nanoparticles in our case is probably favored by a relatively low level of NPs oxidation, which could not strongly affect the degradation kinetics as it took place in the case of deeply oxidized Si NPs^62^,^63^.

Combined together, our results evidence a fast biodegradation of Si-NPs by liver and spleen macrophages into small nanoparticles (<6 nm) or into hydrophilic orthosilicic acid and their rapid renal elimination with the urine (in both cases). In contrast to silica (SiO_2_) nanoparticles^9^,^10^, Si-NPs accumulation did not induce any liver or kidney toxicity, as was confirmed by ALAT, ASAT activities and serum creatinine level, which remained almost unchanged and comparable with those parameters from the control mice group. Here, a slight increase of ALAT 3 h after NPs injection came back to the normal level after 24 h. It should be noted that liver inflammation injury is normally associated with at least 2-fold increase of ALAT activity compared to the basic level^64^, which is obviously not our case. Taking into account this fact, as well as the absence of any histological damage in our experiments, one can conclude that neutrophils infiltration 3 h after NPs injection was not associated with significant hepatic toxicity. In general, the recorded toxicity level looks negligible, especially compared to previously obtained data on silica nanoparticles, which provoked mononuclear inflammatory infiltrate at the portal area and hepatocyte necrosis at the portal triads 1 week after injection^10^, as well as an increase in ASAT level and an elevated number of sinusoidal Kupffer cells 48 h after intravenous administration of rats^10^. Furthermore, Si-NPs accumulation and biodegradation did not cause any significant increase of oxidative stress parameters including catalase, SOD, GPx activities, Vit A and E. These results contrast with the production of ROS generally observed for other types of inorganic nanoparticles and particularly silica nanoparticles^5^,^6^,^7^,^8^,^9^,^10^. Although catalase is specific to reduce hydrogen peroxide (H_2_O_2_), its increased activity at 3 h post administration of Si-NPs is due to the increased H_2_O_2_ level released by infiltrated neutrophils in liver during the inflammation process. In this step NADPH oxidase is activated by forming H_2_O_2_ which is known to exert a toxic effect on pathogenic agents, as reported previously^65^. Finally, the absence of chronic inflammation was confirmed by the transient increase of serum IL-6 level that came back to normal values as soon as 48 h after the Si-NPs administration.

We believe that excellent *in vivo* manifestation of Si-NPs and their stability for 24 h promises a perfect rational design of degradable nanoparticles-based drug delivery systems capable of delivering a therapeutic agent to a target tissue in a closely predictable manner. In addition, laser-synthesized Si-NPs look as very promising candidates for cancer theranostics. The absence of Si-NPs toxicity is obvious since orthosilicic acid is well biocompatible with numerous tissues^3^, while Si naturally exists in humans as a trace element^66^. To the best of our knowledge, our data present the first *in vivo* study, which provides direct evidence of safety of laser-ablated Si-NPs promising a huge potential for biomedical applications with particular emphasis on cancer theranostics.

## Methods

### Synthesis and characterization of Si-NPs

Si-NPs were synthesized by laser ablation of silicon in deionized water, as described in details previously^21^,^37^,^38^. Briefly, a Si target was placed at the bottom of a glass vessel filled with 20 mL of deionized water (18.2 MΩcm). Radiation from Yb:KGW femtosecond laser (Amplitude Systems [Pessac, France], 1025 nm, 480 fs, 500 mJ, 1–5 kHz) was focused with the help of a 750 mm lens onto the target surface to provide the ablation of material. The target was moved at a scanning velocity of 0.35 mm/s in the focusing plane to obtain identical surface conditions during the laser ablation, while the thickness of the water layer above the target was about 1 cm. Such ablation geometry normally leads to a grey coloration of the aqueous solution after 2–5 minutes of the experiment. As the second protocol, we employed methods of two step femtosecond laser fragmentation introduced in a previous study^39^. Ten mL of NPs solution prepared by the first protocol was transferred into a glass cuvette and irradiated, by a focused laser beam of the Yb:KGW femtosecond laser (using the same focusing lens), while the solution was stirred by a magnet to homogenize the ablation process. We used relatively low laser fluence (1 J/cm^2^) to avoid the phenomenon of laser-assisted plasma breakdown of the liquid, but the radiation intensity was high enough to ablate the suspended nanoparticles. After ultracentrifugation at 45000 rpm/min for 25 min, the obtained Si-NPs pellet was dispersed in physiologic sodium chloride solution (NaCl 0.9%). High-resolution transmission electron microscopy (TEM), dynamic light scattering (DLS) and zeta potential measurements were then carried out to characterize properties of laser-synthesized nanoparticles. TEM characterization was done with a JEOL JEM 1,400 system (Peabody, MA, USA). Zeta potential and DLS measurements were performed in a Zetasizer Nano ZS instrument (Malvern Instruments, Orsay, France).

### Cell culture

*In vitro* assays were assessed on normal Human Microvascular Endothelial Cells (HMEC), mouse monocyte-macrophage cells (RAW264.7) and Human Glioblastoma cells (U87-MG cells). The U87-MG cell line was chosen in this study in order to assess future prospects of using Si-NPs for the treatment of glioblastoma, which is in the focus of our current research. HMEC and U87-MG cells were provided by the American Type Culture Collection. RAW264.7 cells were kindly provided by Dr Sarrazin (CIML, Marseille, France). HMEC cells were cultured in MCDB 131 medium containing 10% of heat inactivated fetal bovine serum, 1% (v/v) antibiotics (penicillin–streptomycin), 1% glutamine and 0.1% of epidermal growth factor (EGF). RAW264.7 and U87-MG cells were cultured in DEMEM and EMEM medium respectively, containing 10% of fetal bovine serum, 1% (v/v) antibiotics (penicillin–streptomycin), and 1% glutamine. All cells were kept in a humidified incubator filled with 5% CO_2_ and maintained at 37 °C, as described previously^67^.

### *In vitro* cell survival assay

For cell survival assays, HMEC and RAW264.7 cells were seeded in 96-well plates at 12000 and 5000 cells per well respectively. After 24 h of cells incubation, the culture media were replaced by 150 μ L of corresponding fresh ones containing different concentrations of Si-NPs (0.00, 1.25, 2.50, 5.00, 12.50, 25.00, 50.00 and 100.00 μ g/mL). Following incubation for 72 h, cell viability was determined by MTT 3-(4,5-dimethylthiazol-2-yl)-2,5-diphenyltetrazolium bromide colorimetric assay^67^. This test is based on the reduction of the tetrazolium salt by the mitochondria of living cells. The absorbance measured at 600 nm is proportional to the number of living cells.

### Cells internalization of Si-NPs

The interaction of Si-NPs with cells was studied by TEM. HMEC and U87-MG cells were seeded in 6-well plates at 282000 and 117500 cells per well, respectively. After 24 h of cells incubation, the culture media were replaced by 1.5 mL of corresponding fresh ones containing 50 μ g/mL of Si-NPs for 1, 3, 24, 48 and 72 h. An equivalent cell number, used as control, was exposed to the equivalent culture medium without Si-NPs. After different times of incubation with Si-NPs, the culture media was removed and cells were washed with PBS and fixed with glutaraldehyde 2.5% in 0.1M sodium cacodylate buffer, pH 7.4 for 3 h at room temperature. The cells were then strapped in each well and the pellets were washed three times with 0.1M sodium cacodylate buffer (pH 7.4). Pellets were post-fixed with 2% osmium tetroxide in 0.1M sodium cacodylate buffer for 1h and then dehydrated in series of graded ethanol solutions, filtrated, and embedded in agar 100 epoxy resin, using propylene oxide as a transitional fluid. As a next step, ultrathin sections were cut from dried blocks by a diatom diamond knife on an LBK ultramicrotom Leica UCT and stained with 0.5% aqueous uranyl acetate, followed by Reynold’s lead citrate. Finally, the sections were examined using Tecnai G2 at 200 kV (FEI, Netherdland) TEM and processed by a Veleta camera (Olympus, Japan).

### Animal experimentation

We used a nude mice model, which is frequently used in cancer research, e.g., in tasks of drug delivery and diagnosis. Forty-eight athymic nude female mice (Athymic Nude-Foxn1^*nu*^) (4-weeks-old, 20 ±  2 g) were obtained from the central animal care facilities, Harlan France. Mice were randomly divided into 8 groups of 6 animals and maintained in an air-conditioned room (22–25 °C) on a 12 h light/dark cycle with water and food available. Four groups of 6 athymic nude female mice were intravenously injected in the jugular vein (under isofluorane anesthesia) with a single dose of 20 mg/kg of Si-NPs and then examined for survival 3, 24, 48 h and 7 days after the NPs injection. In contrast, four control groups of athymic nude mice were intravenously injected with NaCl 0.9%. After Si-NPs administration the mice body weight and behavior were monitored to determine the presence or the absence of toxic effects due to the Si-NPs. Animals were sacrificed 3 h, 24 h, 48 h and 7 days after Si-NPs or NaCl 0.9% administration. Twenty-four hours before the sacrifice, animals were placed in individual metabolic cages in order to collect urine and feces. After the treatment, all animal groups were sacrificed under isofluorane anesthesia. Liver, spleen, heart, lungs, kidneys and brain were extracted, washed with NaCl 0.9% at 4 °C, then immerged in liquid nitrogen and stored at − 80 °C until analysis. In addition, urine samples and feces were collected and stored at − 20 °C until analysis.

All experimental procedures and animal care were carried out in accordance with related EU directives, guidelines of the French Government and approved by the Regional Committee for Ethics on Animal Experiments. Experiments and animal care were performed in the “Centre d Exploration Fonctionnelle Scientifique” (CEFOS) authorization number: E 13-055-5) approved by the French Government.

### Transmission electron microscopy of organs

After sacrifice, organ fragments including liver spleen and kidneys, were cut into 1 mm^2^ cubes and fixed by immersion with a 2.5% glutaraldehyde, 2% paraformaldehyde, 2 mM CaCl_2_, 0.1% of tannic acid initially prepared in 0.1 M cacodylate buffer (pH 7.4). 42 Sections of 90 nm were prepared with a diatom diamond knife on an LBK ultramicrotom Leica UCT and stained with 0.5% aqueous uranyl acetate followed by Reynold’s lead citrate. The observation was done with a Tecnai G2 at 200 kV (FEI, Netherdland) and acquire with a Veleta camera (Olympus, Japan).

### Histological analysis

For histological evaluation, organs were excised and then fixed in 5% buffered neutral formalin and embedded in a paraffin wax. 5 μ m sections were cut from each block, and stained primarily with hematoxylin and eosin (H&E) for histopathological study.

### Silicon determination

Biological matrices including liver, spleen, lungs, heart, brain, kidneys, feces and urine were mineralized with nitric acid (4N) and incubated at 100 °C overnight to solubilize all silicon content. The obtained pellets were dissolved in diluted nitric acid (2M) then analyzed by inductively coupled plasma mass spectrometry ICP/MS using a Thermo Series II ICP/MS apparatus (Thermo-Electron, Les Ulis, France) to determine Si content.

### Antioxidant enzymes assay, vitamins A and E

CuZn superoxide dismutase (SOD) activity was determined by the method of Marklund and Marklund^68^ using pyrogallol as the substrate. This method is based on pyrogallol oxidation by the superoxide anion (O_2_^·−^) and its dismutation by the enzyme. One unit (U) of CuZn SOD is defined as the amount of enzyme required to inhibit the rate of pyrogallol auto-oxidation by 50%. Catalase activity was determined by measuring hydrogen peroxide decomposition at 240 nm, as described previously by Beers and Sizer^69^. Glutathion peroxydase (GPx) activity was spectrophotometrically quantified by the decomposition of NADPH as reported by Akerboom and Sies^70^. Vitamins A and E were determined in the serum by HPLC, as described by Le Moel et *al.*^71^.

### Biochemical parameters

Mice blood samples were collected and centrifuged, and the obtained serum was stored at − 20 °C until analysis. The aminotransferase (alanine aminotransferase [ALAT] and aspartate aminotransferase [ASAT]) activities in serum were colorimetrically determined using 2,4-dinitrophenylhydrazine at 505 nm according to kit protocols (Biomaghreb, Tunisia; Randox, United Kingdom). Creatinine level was spectrophotometrically determined using commercial diagnostics kits (Biomaghreb, Tunisia; Randox, United Kingdom). Serum levels of interleukin-6 (IL-6) were measured using a Quantikine ELISA kit (R&D Systems, France).

### Statistics

Data are shown as the mean ±  standard deviation. Comparisons with control were performed by using Student’s test. A value of p **< **0.05 was considered statistically significant.

## Additional Information

**How to cite this article**: Baati, T. *et al.* Ultrapure laser-synthesized Si-based nanomaterials for biomedical applications: in vivo assessment of safety and biodistribution. *Sci. Rep.* **6**, 25400; doi: 10.1038/srep25400 (2016).

## Supplementary Material

Supplementary Information

## Figures and Tables

**Table 1:** Ratio of Si-NPs related to initially intravenously administered dose (20 mg/kg) measured in different organs and urine at different moments after the NPs injection.

Time	% of Si					Total
	Liver	Spleen	Kidneys	Urine	Lungs	
3 h	15.81	3.48	4.02	31.25	5.31	59.88
24 h	24.34	3.07	6.17	47.76	7.85	89.19
48 h	1.01	0.05	1.21	8.08	3.10	13.45
7 days	0.94	0.06	0.27	0.36	0.04	1.67
