# Supplementary material for: Ultrapure laser-synthesized Si-based nanomaterials for biomedical applications: in vivo assessment of safety and biodistribution
Source: Sci Rep. 2016 May 6;6:25400. doi: 10.1038/srep25400 (PMC4858730; doi:10.1038/srep25400)
Supplement: Supplementary Information [file srep25400-s1.pdf]

Supplementary Information

Ultrapure laser-synthesized Si-based nanomaterials for biomedical applications: in vivo assessment of safety and biodistribution

Tarek Baati,[1] Ahmed Al-Kattan,[2] Marie-Anne Esteve,[1,3] Leila Njim,[4] Yury Ryabchikov,[2] Florence Chaspoul,[5] Mohamed Hammami,[6] Marc Sentis,[2,7] Andrei V. Kabashin,[2] Diane Braguer.[1,3]

[1]Aix Marseille Université, INSERM, CRO2 UMR_S911, Faculté de Pharmacie, 27 boul. Jean Moulin, Marseille, France

[2] Aix Marseille Université, CNRS, LP3 UMR 7341, Campus de Luminy, 163 Avenue de Luminy, Case 917, 13288, Marseille Cedex 9, France

[3] Assistance Publique - Hôpitaux de Marseille, Hôpital Timone, 254 rue Saint Pierre, 13385 Marseille, France.

[4] Service d'Anatomie et de Cytologie Pathologique, CHU Monastir 5000, Tunisie.

[5]Aix-Marseille Université, CNRS, UMR 7263, Unité Chimie Physique, Prévention des Risques et Nuisances Technologiques, Faculté de Pharmacie, 13385 Marseille Cedex 5, France

[6] Laboratoire des substances naturelles, Institut National de Recherche et d’Analyse Physicochimique, Sidi Thabet, 2020 Tunisie

[7]Bio-nanophotonics Laboratory, National Research Nuclear University “MEPhI” (Moscow Engineering Physics Institute), 31 Kashirskoe sh., 115409 Moscow, Russia

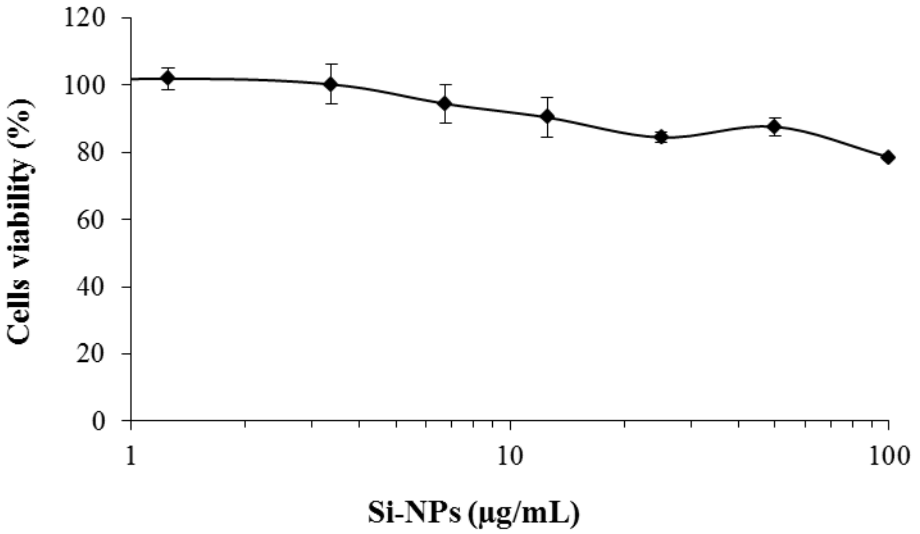

Figure S1: MTT assays of RAW264.7 cells viability following exposure to different concentrations of Si-NPs (1.25- 100 µg/mL) for 72 h. We observed an inhibition of cell survival under 20% up to high concentrations of Si-NPs (100 µg/mL),

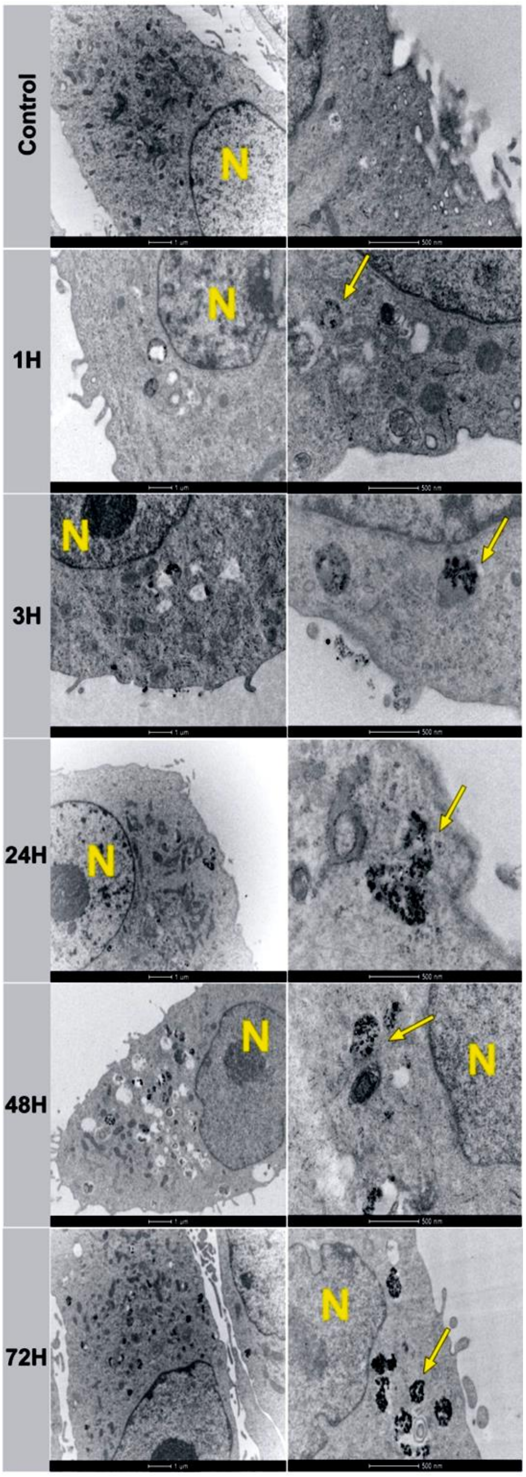

Figure S2: TEM image of U87-MG cells showing kinetics of Si-NPs cell internalization studied 1, 3, 24, 48 and 72 h after the incubation with 50 µg/mL of Si-NPs. The nanoparticles are visible inside lysosomes (arrows) and the amount increased progressively in a time-dependent manner.

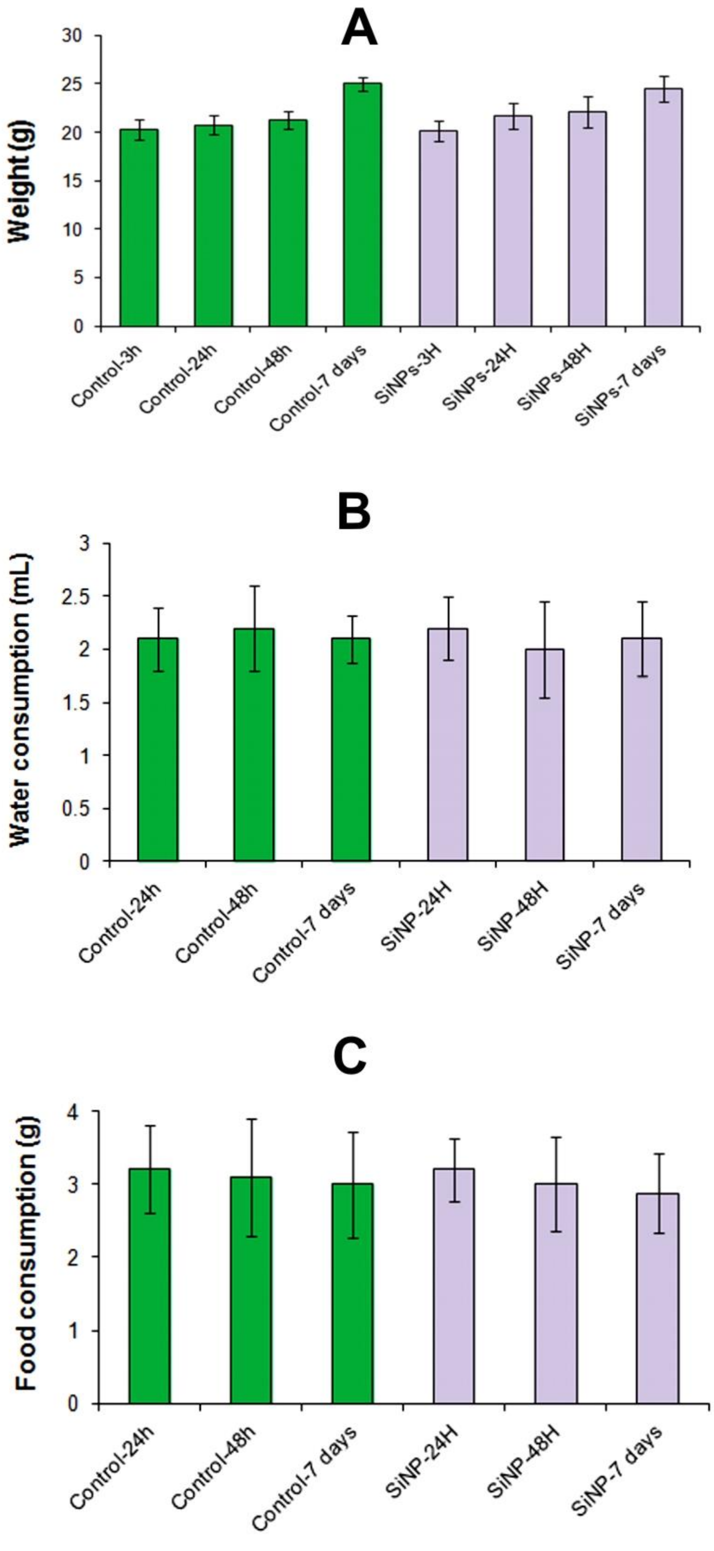

Figure S3: **(A)** Evolution of the mean body weight of mice measured during 3 h, 24 h, 48 h and 7 days after intravenously administration of 20 mg/kg of Si-NPs. **(B)** Water consumption, **(C)** Food consumption. Results showed normal corporal weight increase with no statistically significant differences between Si-NPs treated and control groups ($p < 0.05$). No statistically significant differences were monitored in food consumption or water intake.

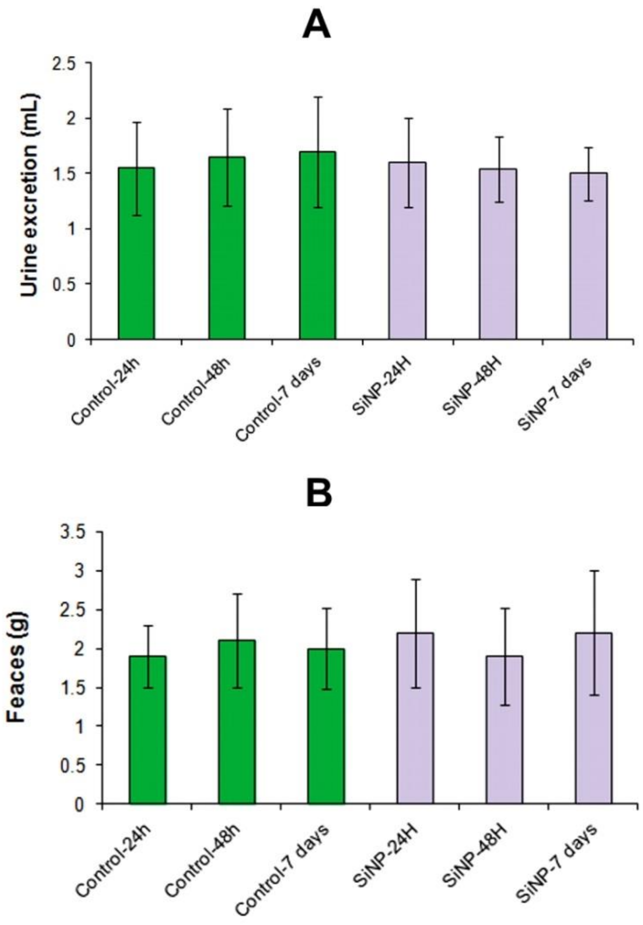

Figure S4: (A) urine excretion and (B) dejection excretion of control and treated mice administered intravenously with 20mg/kg of Si-NPs after 3 h, 24 h, 48 h and 7 days. No statistically significant differences were monitored in urine volume or dejection weight between treated and control groups ($p < 0.05$).

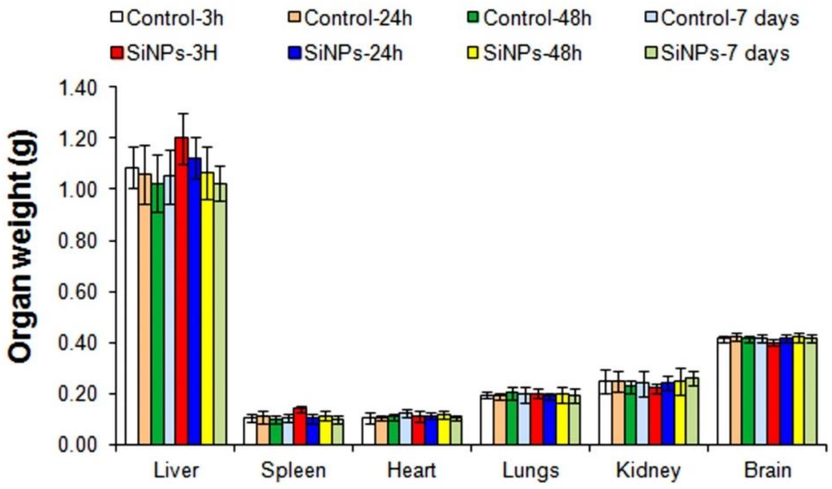

Figure S5: Organ weight of different mice groups sacrificed at 3 h, 24 h, 48 h and 7 days after intravenously administration of 20 mg/kg of Si-NPs. Organ weight comparison between treated and control groups did not show significant statistically difference ($p < 0.05$).

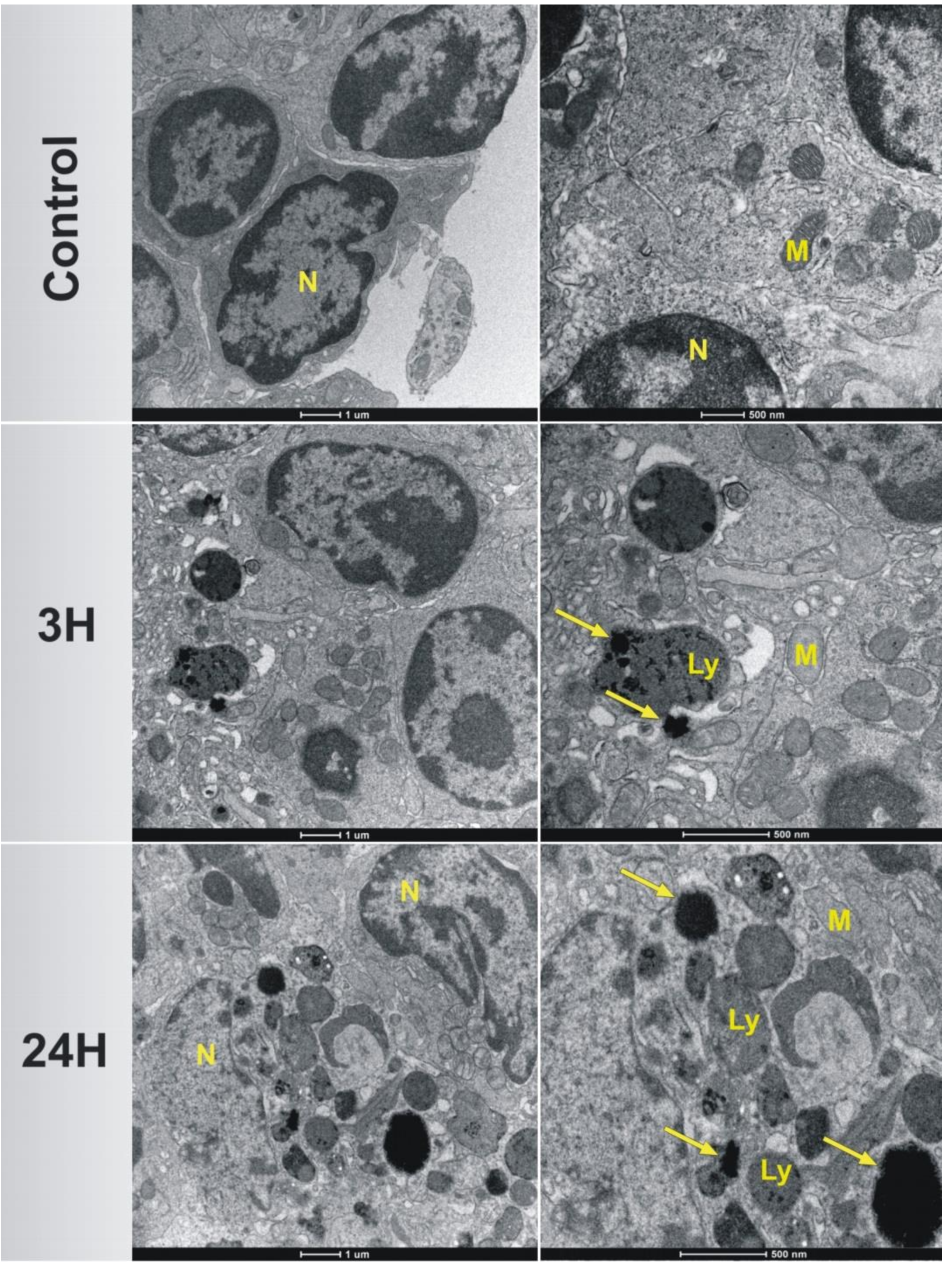

Figure S6: TEM images of spleen 3 h and 24 h after the intravenous injection of Si-NPs (20 mg/kg) compared to the control group. Arrows show the presence of electron dense Si-NPs within the lysosomes (Ly) in macrophages.

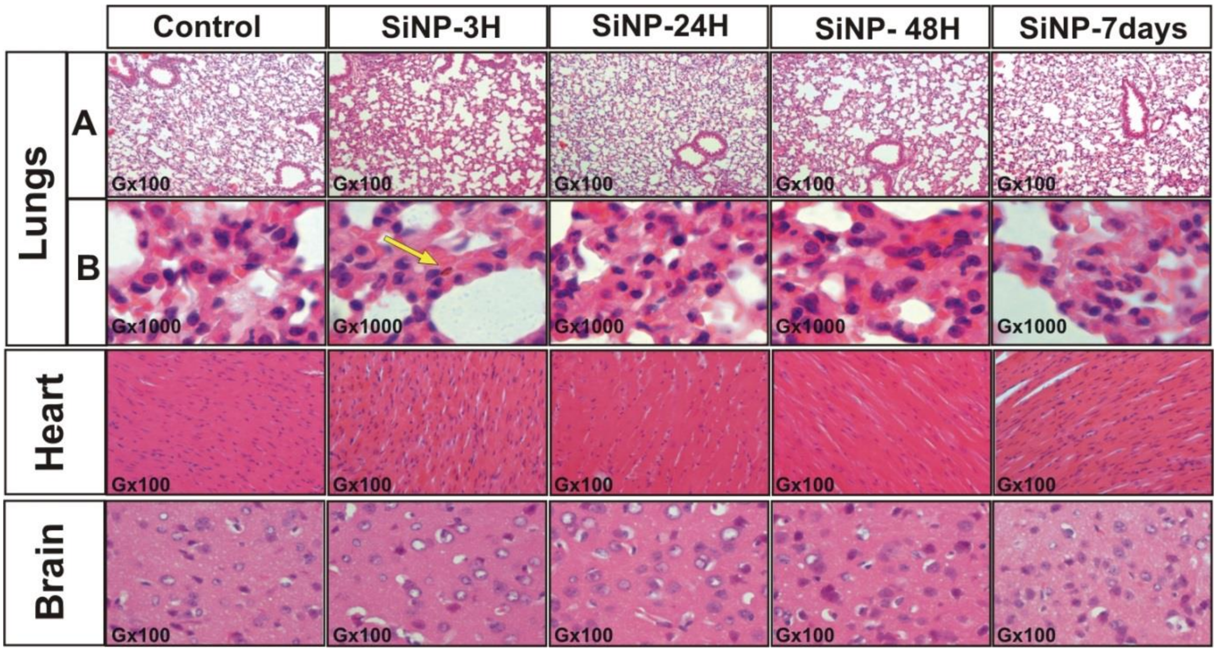

Figure S7: Histological sections of lungs, heart and brain 3 h, 24 h, 48 h and 7 days after the intravenous administration of Si-NPs in comparison with the control group. All sections were stained with hematoxylin–eosin. Histological examination of these organs did not reveal any histological alteration or accumulation of Si-NPs. (B) is the magnification of (A). Occasional accumulation of Si-NPs inside pulmonary capillary after 3 h was observed, as shown by arrow, but this phenomenon was not accompanied by any substantial toxicity effect.

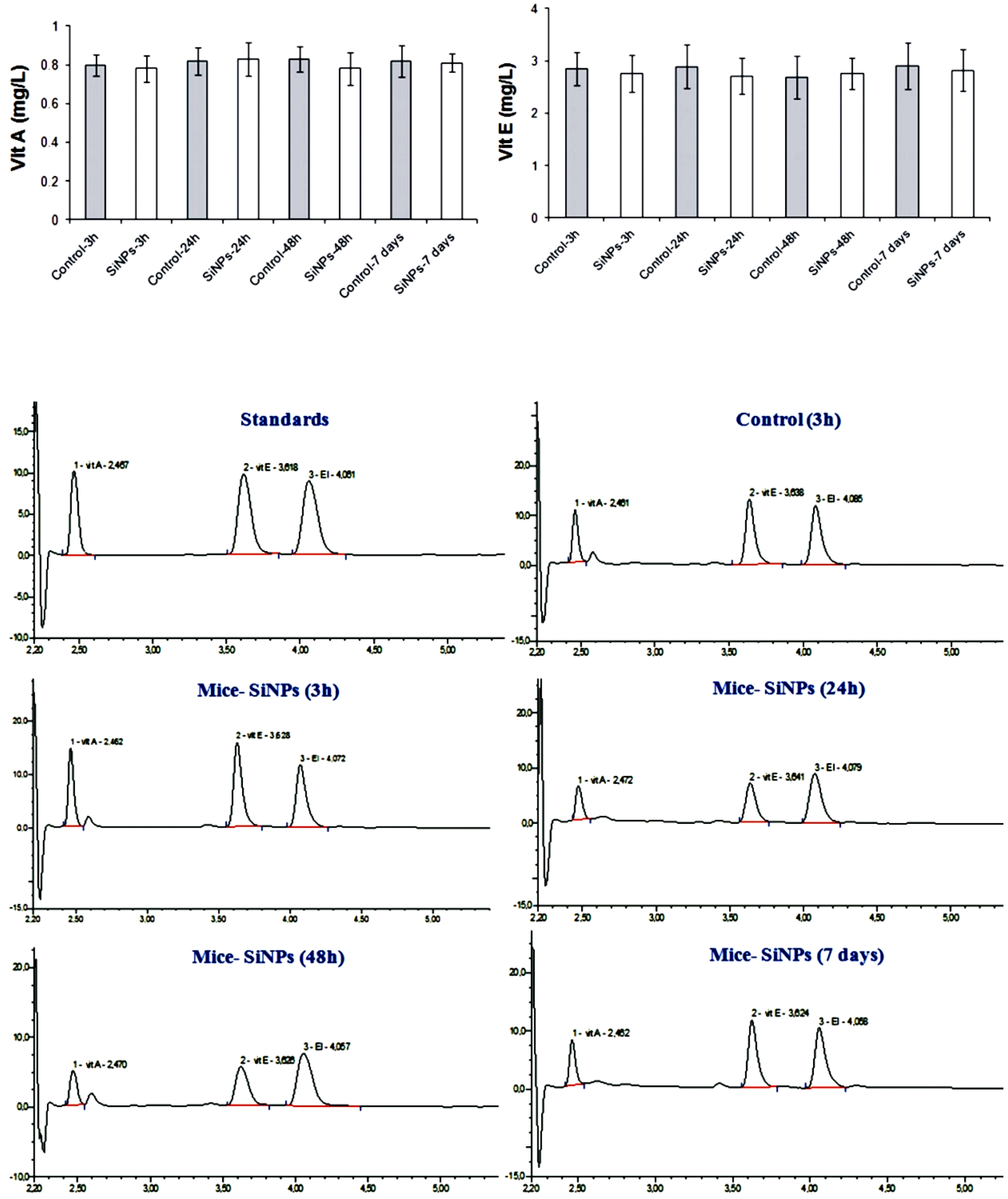

Figure S8: (Top) Serum vitamins A and E levels determined 3h, 24h, 48h, and 7 days after the intravenously administration of SiNPs (20mg/kg) compared to the control group (n=6, data are the mean ± SD). The determination of Vit A and E in all treated animals did not show any significant difference compared to the control groups. **(Down)** Chromatograms of standards (Vit A (retinol), Vit E (α-tocopherol) and internal standard (ester of α-tocopherol prepared in ethanol)), and serum extracts of control and treated mice 3 h, 24 h, 48 h, and 7 days after the intravenously administration of SiNPs (20 mg/kg). UV-visible detection was recorded at 290 nm. Retention times of Vit A, Vit E and internal standard (EI) = 2.46 min, 3.62 min and 4.07 min respectively.

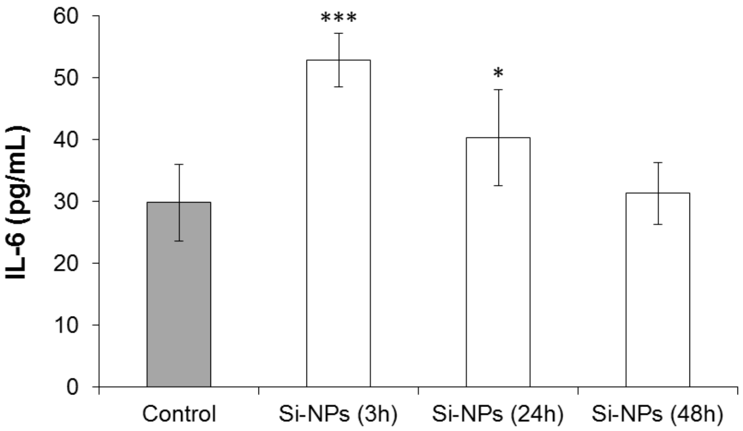

Figure S9: Serum Interleukin-6 level 3 h, 24 h, and 48 h after the intravenous administration of Si-NPs (20 mg/kg). (n=4, data are the mean ± SD, and statistical significance was determined by Student's t-test (* $p < 0.05$, *** $p < 0.001$).
